# Epidemiology of Glomerular Disease in Southern Arizona: Review of 10-Year Renal Biopsy Data

**DOI:** 10.1097/MD.0000000000003633

**Published:** 2016-05-06

**Authors:** Sangeetha Murugapandian, Iyad Mansour, Mohammad Hudeeb, Khaled Hamed, Emad Hammode, Babitha Bijin, Sepehr Daheshpour, Bijin Thajudeen, Pradeep Kadambi

**Affiliations:** From the Department of Nephrology (SM, BB, BT, PK); and Department of Medicine (IM, MH, KH, EH, SD), University of Arizona Medical Center, Tucson, AZ.

## Abstract

Glomerulonephritis stands third in terms of the etiologies for end-stage kidney disease in the USA. The aim of this study was to look at the patterns of biopsy-proven glomerulonephritis based on data from a single center.

Kidney biopsy specimens of all patients above the age of 18 years, over a 10-year period, who had diagnosis of nondiabetic glomerular disease, were selected for the study.

The most common histopathological diagnosis was focal and segmental glomerulosclerosis (FSGS) (22.25%, 158/710) followed by membranous nephropathy (20.28%, 144/710) and immunoglobulin (Ig)A nephropathy (19.71%, 140/710). There was male preponderance in all histological variants except IgA nephropathy, lupus nephritis, and pauci-immune glomerulonephritis. The race distribution was uneven, and all histological variants, except minimal change disease and lupus nephritis, were more commonly seen in whites. In a separate analysis of the histological pattern in Hispanics, lupus nephritis was the most common pathology (28.70%, 62/216) followed by FSGS (18.05%, 39/216). In American Indian population, the most common pathology was IgA nephropathy (33.33%, 8/24) followed by FSGS (16.67%, 4/24).

This study highlights the histopathological patterns of glomerular disease in southern Arizona. The data suggest regional and ethnic variations in glomerular disease that may point towards genetic or environmental influence in the pathogenesis of glomerular diseases.

## INTRODUCTION

Glomerulonephritis (GN) is responsible for 16% of all causes of end-stage kidney disease (ESKD) in the United States of America (USA).^1^ Knowledge about the incidence and prevalence of GN and its regional trends are mandatory for nephrologists to adopt measures for preventing patients with glomerular disease from progression to ESKD. But at the same time, the prevalence of glomerular diseases in the general population is hard to evaluate because optimal conditions for performing epidemiologic surveys are difficult to find. Moreover, a glomerular disease registry does not exist in most states. A review of renal biopsy data may provide an insight into the spectrum of GN prevalent within a particular community or geographic area. There are no valid data about the epidemiology of GN reported in southern Arizona. This study aims at looking at the pattern of glomerular disease in this region of Arizona.

## METHODS

This was a retrospective study from a single pathology laboratory at the Banner University Medical Center, Tucson, Arizona. Our pathology laboratory receives referrals from other institutions in southern Arizona including the Veterans Affairs hospital. This lab analyzes the greatest number of renal biopsies in the region and is the only laboratory in southern Arizona where immunofluorescence (IF) and electron microscopy (EM) are routinely performed. EM was not done in some of these specimens due to nonavailability at that time.

We analyzed renal biopsy specimens from patients above the age of 18 years with glomerular renal disease performed during the period 2004 to 2014. Kidney transplant biopsies and biopsies showing diabetic nephropathies were excluded. Histological diagnoses were grouped into one of the following 8 categories: minimal change disease (MCD), focal and segmental glomerulosclerosis (FSGS), membranous nephropathy (MGN), membrano-proliferative GN (MPGN), pauci-immune (PI) GN, immunoglobulin (Ig)A nephropathy (IgAN), lupus nephritis, and others. When 2 histopathological patterns were found in 1 and the same pathology specimen, predominant pathology was selected as the histopathological diagnosis. The following demographic parameters were also systematically collected from the medical records archive: sex, age, and race. This study was approved by Institutional Review Board of the University of Arizona (protocol number 1405316119).

### Statistical Analysis

Descriptive statistics are used to present the distribution of the histological types of glomerulopathies and their relative frequencies. Age data are presented as means ± standard deviations (SDs). The data are analyzed using the Statistical Package for the Social Sciences (SPSS) software, version 11.5.

## RESULTS

A total of 710 biopsy specimens were chosen for the study. There were 371 females and 339 males (ratio of 1.09:1). The racial breakup of the study population was as follows: Whites constituted 35.91% (255/710), Hispanics 30.4% (216/710), African American 7.32% (52/710), American Indian 3.38% (24/710), and others (Asians/undeclared) 22.95% (163/710). The racial profile of the population of southern Arizona is as follows: White American 40% to 42%, Hispanics 48% to 50%, African American 3% to 4%, American Indian 2% to 3%. The sex and racial distribution of the study population is depicted in Table 1.

**TABLE 1:**
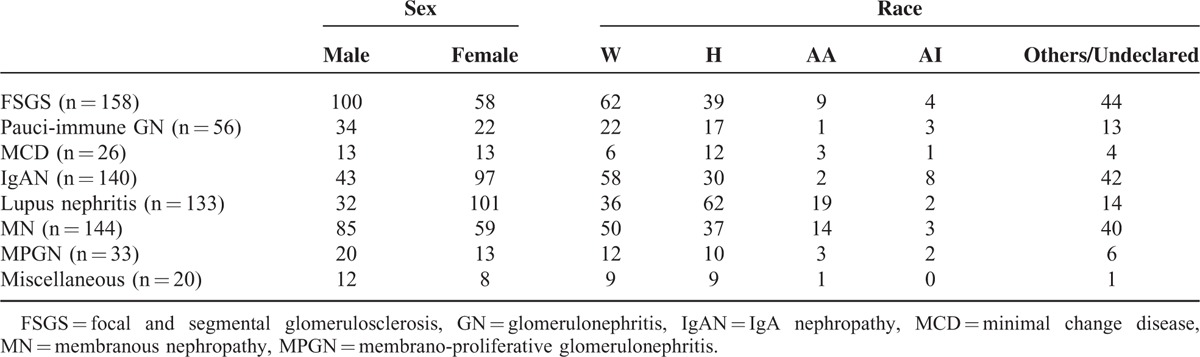
Showing the Sex and Racial Distribution of Study Population

**FIGURE 1:**
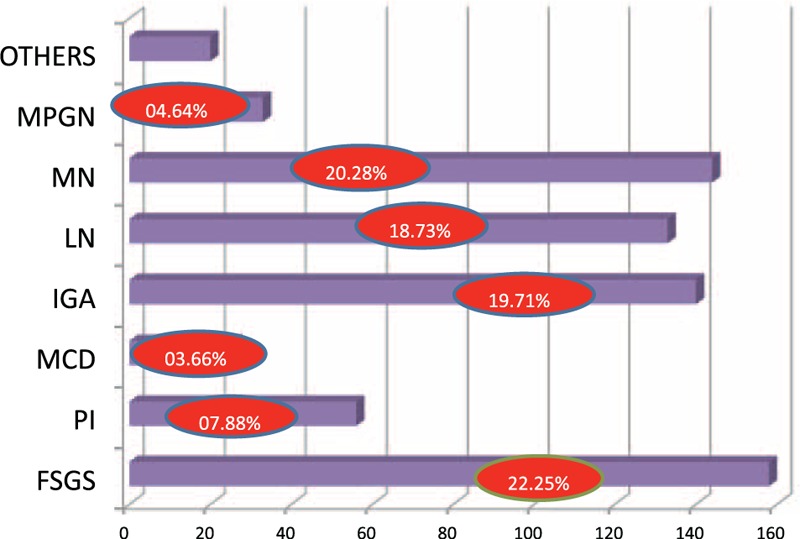
Bar diagram showing frequency of glomerular diseases. Percentage of each glomerular disease represented in red circles. FSGS = focal and segmental glomerulosclerosis, IgAN = IgA nephropathy, LN = lupus nephritis, MCD = minimal change disease, MN = membranous nephropathy, MPGN = membrano-proliferative glomerulonephritis, PI = pauci-immune glomerulonephritis.

**FIGURE 2:**
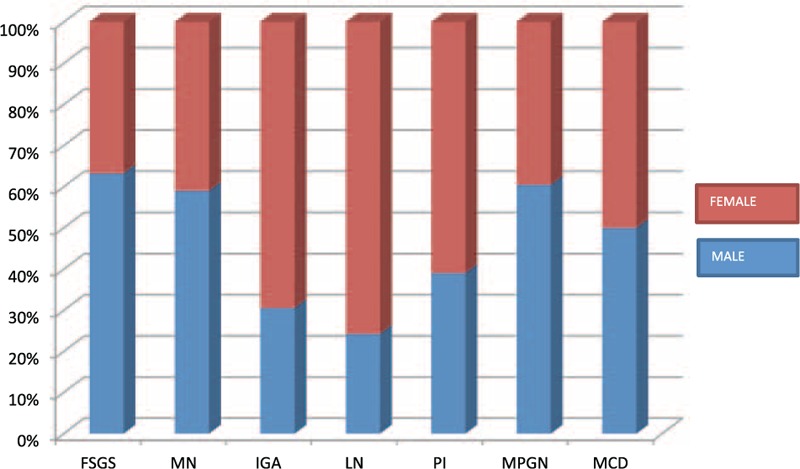
Bar diagram showing male, female distribution of each glomerular disease. FSGS = focal and segmental glomerulosclerosis, IgAN = IgA nephropathy, LN = lupus nephritis, MCD = minimal change disease, MN = membranous nephropathy, MPGN = membrano-proliferative glomerulonephritis, PI = pauci-immune glomerulonephritis.

**FIGURE 3:**
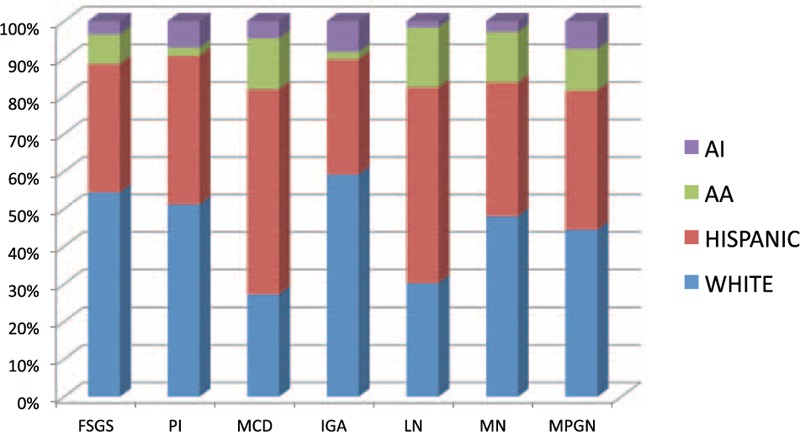
Bar diagram showing racial distribution of each glomerular disease. AA = African American, AI = American Indian, FSGS = focal and segmental glomerulosclerosis, IgAN = IgA nephropathy, LN = lupus nephritis, MCD = minimal change disease, MN = membranous nephropathy, MPGN = membrano-proliferative glomerulonephritis, PI = pauci-immune glomerulonephritis.

The nondiabetic glomerulopathies (including both primary and secondary glomerular diseases) in order of frequencies were FSGS (158 cases; 22.25%), membranous GN (144 cases; 20.28%), immunoglobulin A (IgA) nephropathy (140 cases; 19.71%), lupus nephritis (133 cases; 18.73%), pauci-immune GN (56 cases; 7.88%), MPGN (33 cases; 4.64%), and MCD (26 cases; 3.66%) (Figure 1). Other diagnosis included postinfectious GN (8 cases; 1.12%), anti-GBM disease (3 cases; 0.4%), amyloidosis (2 cases; 0.2%), fibrillary GN (2 cases; 0.2%), and cryoglobulinemic GN (2 cases; 0.2%). There was 1 case each of C3 GN, nodular glomerulosclerosis, and collapsing GN. The sex and racial distribution of each glomerulopathy are represented in Figures 2 and 3.

### Focal and Segmental Glomerulosclerosis

Focal and segmental glomerulosclerosis was the most common histopathological diagnosis encountered (22.25%). Mean age of the group was 54 ± 19.09 years. Most cases of FSGS were seen in Whites (39.24%). Males dominated females with a ratio of 1.72:1.

### Membranous Glomerulonephritis

Membranous nephropathy was the second most common histopathological diagnosis (20.28%). Mean age of the group was 49.82 ± 19.78 years. Most cases were seen in Whites (34.72%). The ratio of males to females was 1.44:1.

### IgA Nephropathy

IgA nephropathy was seen in 19.71% of the biopsies. Mean age of the group was 41.75 ± 19.09 years. Most cases were seen in Whites (41.42%). It was seen predominantly in females with ratio 2.25:1.

### Lupus Nephritis

Lupus nephritis was seen in 18.73% of the biopsies. Mean age of the group was 34.86 ± 18.99 years. Most cases were seen in Hispanics (46.61%). Lupus nephritis was seen predominantly in females, with ratio of females to males of 3.15:1. Class 4 lupus nephritis was the most common (50.37%).

### Pauci-immune Glomerulonephritis

Pauci-immune GN constituted 7.88% of the biopsies. Mean age of the group was 59.56 ± 9.46 years. Most cases were seen in Whites (39.28%). Females dominated over males with ratio of 1.54:1.

### Membrano-proliferative GN

Membrano-proliferative GN constituted 4.64% of the biopsies. Mean age of the group was 54.39 ± 13.34 years. Most cases were seen in Whites (36.36%). Males dominated over females, with ratio of 1.53:1.

### Minimal Change Disease

Minimal change disease constituted 3.66% of the biopsies. Mean age of the group was 45.88 ± 9.19 years. Most cases were seen in Hispanics (46.15%). Male to female ratio was 1:1.

### Glomerulonephritis in Hispanics

**FIGURE 4:**
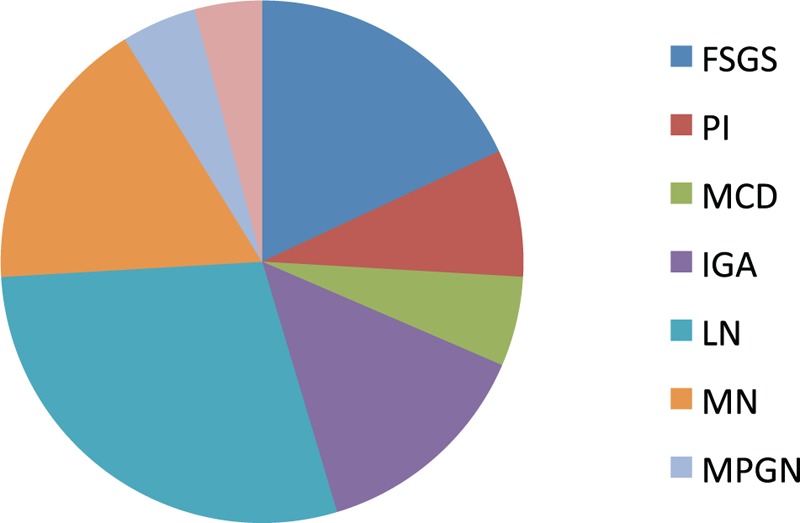
Pie chart showing frequency of each glomerular disease in Hispanics. FSGS = focal and segmental glomerulosclerosis, IgAN = IgA nephropathy, LN = lupus nephritis, MCD = minimal change disease, MN = membranous nephropathy, MPGN = membrano-proliferative glomerulonephritis, PI = pauci-immune glomerulonephritis.

Out of the 216 Hispanic patients, there were 116 females and 100 males. The mean age of the group was 41.12 ± 16.97 years. Lupus nephritis was the most common histopathology (28.70%) followed by FSGS (18.05%) and membranous nephropathy (17.12%) (Figure 4).

### Glomerulonephritis in Native Americans (American Indians)

**FIGURE 5:**
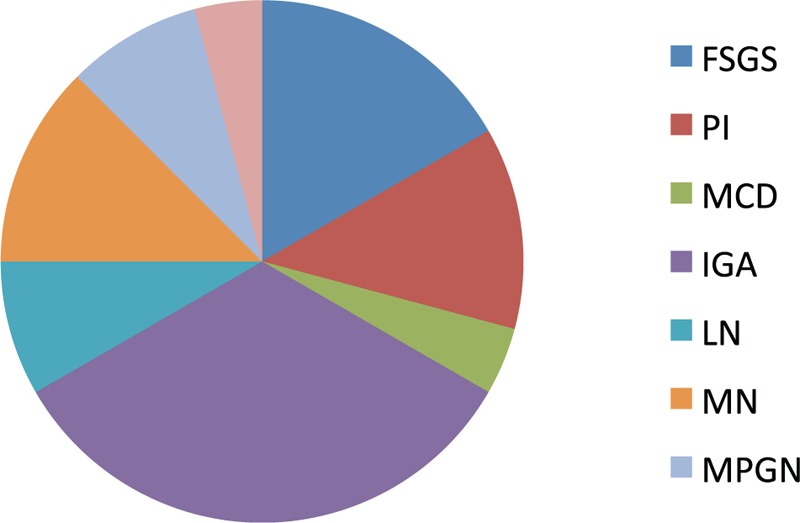
Pie chart showing frequency of each glomerular disease in American Indians. FSGS = focal and segmental glomerulosclerosis, IgAN = IgA nephropathy, LN = lupus nephritis, MCD = minimal change disease, MN = membranous nephropathy, MPGN = membrano-proliferative glomerulonephritis, PI = pauci-immune glomerulonephritis.

Out of the 24 American Indians, there were 11 females and 13 males. The mean age of the group was 50.91 ± 2.82 years. IgA nephropathy was the most common histopathology (33.33%) followed by FSGS (16.67%). Membranous nephropathy and pauci-immune GN were seen in 12.5% each (Figure 5).

## DISCUSSION

Studying the epidemiological aspects of renal diseases, both primary and secondary, may help to identify the frequency of glomerulopathy or other kidney diseases. It may also help in investigating the determinants such as ethnic, environmental, or genetic factors contributing towards disease development, the potential regional differences, the local biopsy indications, and other relevant clinical and histological features. This study gives the epidemiologic information of nondiabetic glomerular diseases in our population and can form the basis for further studies. There is no similar study to our knowledge which has looked at the prevalence of GN in a region with racial distribution similar to ours.

The high prevalence of FSGS in this population is supported by observations from other investigators. An increasing incidence of FSGS has been noted by D’Agati et al and Hass et al.^2^ Kitiyakara et al reported that ESKD secondary to FSGS has increased 11-fold in the past 2 to 3 decades.^2^ These studies showed an increasing incidence of FSGS in white, and also black adults. Since the African American population in the area was less than 5%, we will not be able to generalize this finding to our population. But at the same time, we can assume that the frequency of FSGS is high in whites in this region. Factors like obesity or unidentified environmental factors might be responsible for high frequency of FSGS in this population. This finding raises the question whether the higher frequency reported for FSGS is simply due to better renal biopsy specimens or improvement in diagnostic modalities including histopathological examination. But at the same time, because of lack of EM in some cases, we did not try to make a distinction between primary and secondary FSGS. There might have been cases of familial FSGS information which we could not reveal due to lack of information about medical history and genetic analysis.

Another interesting observation in this study was the finding of lupus nephritis being the most common histopathological pattern observed in Hispanics. Lupus nephritis is seen mostly in the uninsured, low socioeconomic status minorities, especially in the southern USA.^3^ Studies have also shown that European ancestry may be protective against the development of lupus nephritis, which might also explain lower incidence of LN in whites compared to Hispanics.^4^ LUMINA, a multiethnic US cohort study found that LN occurs more frequently seen in Hispanics and African Americans.^5^ Several other studies have reported a higher frequency of LN in Hispanics compared with Caucasians. This association is linked to genetic factors which confer higher susceptibility of Hispanics to lupus. The major ones described in literature include greater frequency of expression of HLA DR3, DR2 subtype DRB1^∗^ 1501, and ITGAM gene. Other associations include mutation of IRF5 gene and STAT4 gene.^6^

In the American Indian population, the most common nondiabetic GN was IgA nephropathy. This was substantiated by other studies as well. Studies of the Zuni Pueblo reported high rates of mesangiopathic GN, predominantly IgA. A biopsy series from Zuni people with nondiabetic kidney disease included 77% with mesangio-proliferative GN.^7^ A series of 166 American Indian renal biopsy specimens from 1971 to 1989 showed a very high proportion with mesangial proliferative GN with mesangial immunoglobulin deposition.^8^ Smith and Tung^9^ looked at the frequency of IgAN in native Indians based on renal biopsy and found that that IgAN was found in 38%. Whether the high prevalence is due to immunogenetic factors or high frequency of renal biopsy for abnormal urinary findings is not known at this point (because of the high prevalence of diabetes mellitus among American Indians, renal injury in most patients is attributed to diabetes and hence they do not get biopsied unless they have abnormal urinary findings). It could also be due to high prevalence of alcohol use in this population resulting in liver diseases.^10^

In this study population, the frequency of IgAN was more common in females with a ratio of 2.25:1. IgAN is a disease with male predominance in USA with a ratio of almost 6:1.^11^ A similar female preponderance was seen in some of the studies reported from Southeast Asia including China and Japan, where IgAN is the most common primary glomerular disease.^12,13^ To our knowledge, such a finding was never reported in any other study in USA. The other diseases with female preponderance were lupus nephritis and pauci-immune GN. Pauci-immune GN is considered as a GN without any sex predilection.^14^ But in this population, the females dominated males in the ratio 1.54: 1. Similar findings were noted in studies by Jayne et al^15^ and De Groot et al.^16^ Although lupus is common in females, renal involvement from lupus is seen to be more common in males compared with females in some studies.^17,18^ But in this population, lupus nephritis was found to be more common in females. The reason of this female preponderance in all these disease types is not clear at this point and it needs further study.

In this study, Hispanics were relatively younger compared with Caucasians. This is an important observation considering the increasing incidence of chronic kidney disease (CKD) and ESKD in Hispanics.^19^ Although glomerular disease accounts for only 9% of the ESRD in Hispanics, this is essentially a preventable cause for ESKD compared with diabetes mellitus or hypertension, which account for almost 75% of the causes of ESKD in Hispanics.^19^ It is also notable that the glomerular disease with youngest age predilection was lupus nephritis, which was also the most common glomerular disease in Hispanics. Screening for glomerular disease, especially lupus nephritis, should be intensified in this population. Early recognition and treatment of glomerular diseases may decrease the number of patients progressing to dialysis and hence reduce the economic burden. This has already been proved in observational studies.^20^ The current screening guidelines for lupus nephritis published by American College of Rheumatology have adopted few concrete steps in this direction.^21^ But at the same time, there are still no set guidelines for frequency of monitoring for lupus nephritis in patients with systemic lupus erythematosus (SLE) and patients with early lupus nephritis (class I and II).

Pauci-immune GN was found to have frequency of 7.88% among all biopsies. There are not much data on the incidence and prevalence of pauci-immune GN in the USA. Data on crescentic GN show that pauci-immune accounts for 60% to 80% of all crescentic GNs .^22^ In the study by Swaminathan et al,^2^ during the period 1974 to 2003, crescentic GN accounted for 5.12% of all GN cases. This means that the actual frequency of pauci-immune GN might have been between 3% and 4%, which is much lower than what is reported in our study. The reason for the higher frequency seen in our study is speculative rather than evidence. When we tried to analyze pauci-immune GN and anti-neutrophil cytoplasmic antibodies (ANCA) prevalence, out of all types (ANCA-positive and ANCA-negative) p-ANCA-positive pauci-immune GN accounted for more than 50%. Literature shows that microscopic polyangitis (mostly a p-ANCA-related vasculitis) is seen mostly in warmer climates.^23^ Because of geographic features, southern Arizona has a warmer climate, which might explain the high frequency of pauci-immune GN in this region. The role of other environmental factors like infection and silica needs further studies.

This study has several drawbacks. This is a single-center, retrospective study, and hence the finding may not be applicable to the entire population of USA. It only gives the frequency of each glomerular disease that was referred to our center. In 30% to 40% of the cases, we reviewed only the histopathology specimen and a brief clinical history accompanied the request without giving details of protein quantification or creatinine value. Since detailed clinical information was lacking in those cases, we decided not to report the indications for renal biopsy. Additionally, the observed differences in glomerular pattern should be interpreted with caution, since they may be influenced by screening procedures for detecting asymptomatic patients, selective referral of patients with progressive renal disease to nephrological centers, and indication for renal biopsy.

## CONCLUSIONS

This study based on regional database has enabled us to ascertain the most frequent GN in our region. It has also helped in creating a database which can become the basis for future studies. Although the study does not give an estimate of the actual incidence or prevalence, it does shed light on the differences in trend in glomerular diseases in southern Arizona compared with other parts of USA. It also highlights the point that epidemiology of glomerular diseases cannot be generalized and each region of the nation should have its own database to see the actual trend. This might help in managing this disease entity more effectively by identifying the factors predisposing to glomerular diseases. We also need more well designed studies to investigate the regional variations in incidence and prevalence of glomerular disease.
